# The endosomal protein sorting nexin 4 is a synaptic protein

**DOI:** 10.1038/s41598-020-74694-6

**Published:** 2020-10-26

**Authors:** Sonia Vazquez-Sanchez, Miguel A. Gonzalez-Lozano, Alexarae Walfenzao, Ka Wan Li, Jan R. T. van Weering

**Affiliations:** 1 Department of Functional Genomics, Center for Neurogenomics and Cognitive Research, Amsterdam Neuroscience, VU University, Amsterdam, The Netherlands; 2 Department of Molecular and Cellular Neurobiology, Center for Neurogenomics and Cognitive Research, Amsterdam Neuroscience, VU University, Amsterdam, The Netherlands; 3 Clinical Genetics, Center for Neurogenomics and Cognitive Research, Amsterdam Neuroscience, Amsterdam UMC Location VUmc, De Boelelaan 1085, 1081 HV Amsterdam, The Netherlands

**Keywords:** Cell biology, Membrane trafficking, Endosomes, Cellular neuroscience, Synaptic transmission

## Abstract

Sorting nexin 4 (SNX4) is an evolutionary conserved protein that mediates recycling from endosomes back to the plasma membrane in yeast and mammalian cells. SNX4 is expressed in the brain. Altered protein levels are associated with Alzheimer’s disease, but the neuronal localization and function of SNX4 have not been addressed. Using a new antibody, endogenous neuronal SNX4 co-localized with both early and recycling endosome markers, similar to the reported localization of SNX4 in non-neuronal cells. Neuronal SNX4 accumulated specifically in synaptic areas, with a predominant localization to presynaptic terminals. Acute depletion of neuronal SNX4 using independent short hairpin RNAs did not affect the levels of the transferrin receptor, a canonical SNX4 cargo. Quantitative mass spectrometry revealed that upon SNX4 knockdown the class of proteins involved in neurotransmission was the most dysregulated. This included integral membrane proteins at both the presynaptic and postsynaptic side of the synapse that participate in diverse synaptic processes such as synapse assembly, neurotransmission and the synaptic vesicle cycle. These data suggest that SNX4 is implicated in a variety of synaptic processes.

## Introduction

Sorting nexin 4 (SNX4) is an evolutionary conserved protein that mediates endosomal recycling from endosomes back to the plasma membrane^1,2^. SNX4 is a member of the sorting nexin (SNX) family characterized by a phosphatidylinositol 3-phosphate binding domain (phox homology (PX) domain)^3^, which is necessary for peripheral membrane localization^4,5^. More specifically, SNX4 is part of the SNX- Bin/Amphiphysin/Rvs (BAR) subfamily characterized by a carboxy-terminal BAR domain that binds to curved membranes upon dimerization^4,6^. SNX4 forms tubules that emanate from endosomes during the RAB5-RAB7 (early endosome to late endosome) and RAB4-RAB11 (early recycling endosome to endosome recycling compartment) transition^7^. Hettema et al.^8^ showed that silencing the yeast homologue of SNX4 (Snx4p) decreases the levels of Scn1p (an exocytic v-SNARE) at the plasma membrane and increases Scn1p degradation by the vacuole (the yeast equivalent of the mammalian lysosome)^8^. In HeLa cells, a similar SNX4 pathway has been observed: SNX4 recycles the transferrin receptor (TFNR), an iron-transporting receptor, from endosomes to the plasma membrane thereby avoiding its lysosomal degradation^9^.

SNX4 is expressed in the brain and SNX4 protein levels are decreased by 70% in brains of severe Alzheimer’s disease (AD) cases^10^. Beta-secretase 1 (BACE-1) is an enzyme involved in proteolytic processing of the amyloid precursor protein, which leads to the formation of the pathological amyloid-β (Aβ) peptide in AD^11^. Two recent studies indicated that SNX4 dysregulation mistargets BACE-1 to late endosomal compartments, which impacts Aβ production^10,12^. Hence, neuronal SNX4 dysregulation might be involved in Aβ production and AD aetiology.

While SNX4 has been associated with pathological mechanisms in the brain, the physiological role and subcellular distribution of SNX4 in neurons is currently unknown. Here, we characterized the localization of endogenous SNX4 in primary mouse neurons using a new antibody. Endogenous SNX4 partially co-localized with both early and recycling endosome markers, which is in accordance with the previously established role of SNX4 in non-neuronal cells. In contrast to non-neuronal cells, neuronal SNX4 depletion did not decrease the levels of the canonical SNX4 cargo TFNR^9^. Neuronal SNX4 accumulated specifically in synaptic areas, with a predominant localization to presynaptic terminals. Based on the dysregulation of membrane proteins in synaptic terminals measured by quantitative mass spectrometry in SNX4 knockdown neurons, we propose that SNX4-dependent endosomal sorting is functionally relevant for synaptic terminals.

## Results

### Novel SNX4 antibody specifically labels endogenous mouse SNX4 in western blot and immunocytochemistry

**Figure 1:**
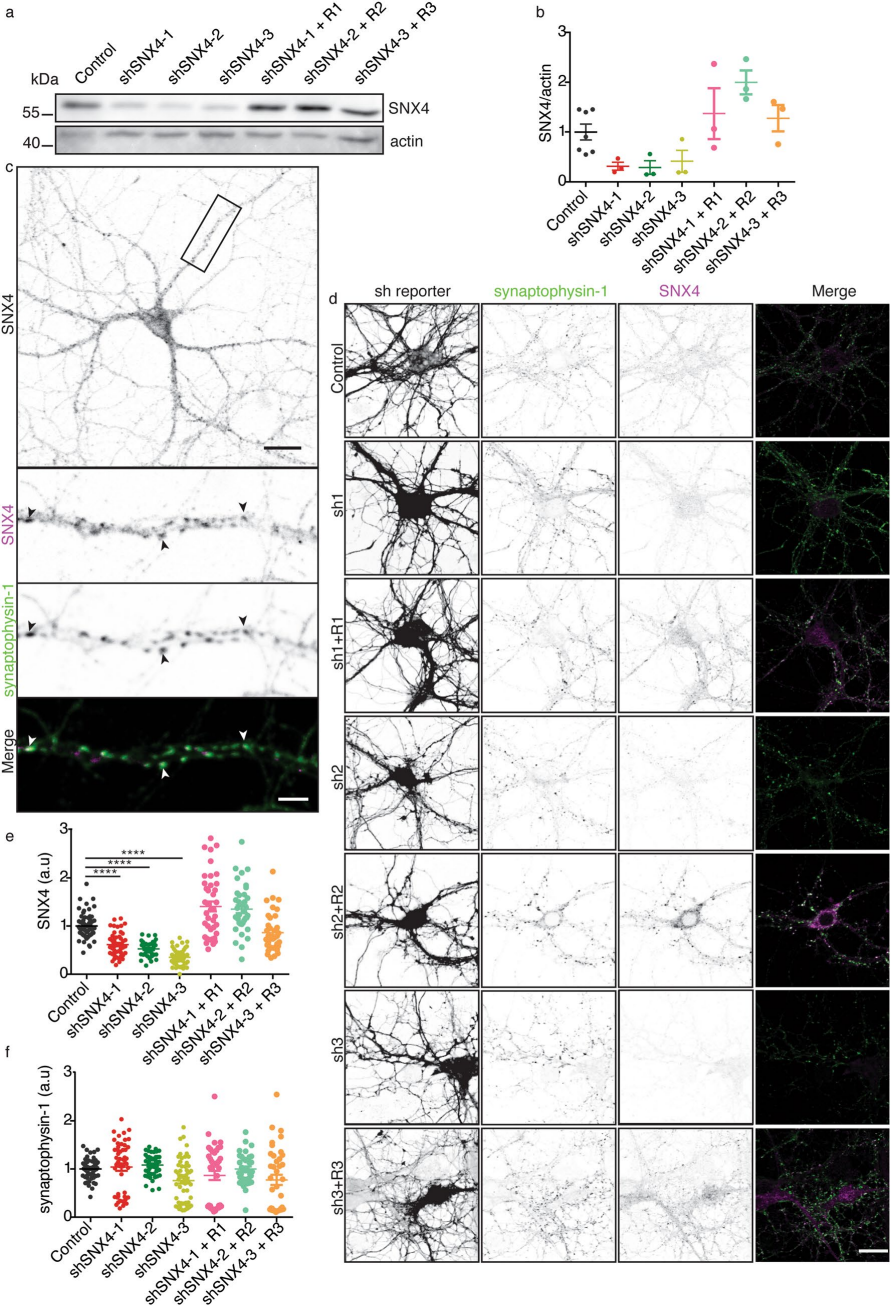
Novel SNX4 antibody detects endogenous mouse SNX4 by western blot and immunocytochemistry. (**a**) Representative cropped SNX4 and actin western blot of control neurons, neurons transfected with shRNAs against SNX4 and neurons transfected with the shRNA and corresponding rescue construct (description of the constructs in materials and methods and Supplementary Fig. S1). Original uncropped SNX4 and actin blot are shown in Supplementary Fig. S1. (**b**) Quantification of SNX4 levels normalized to actin in western blot. Values are presented as a ratio compared to the control condition. (N = 3 blots/cultures). (**c**) Confocal microscopy image of neurons immunolabelled with SNX4 and synaptophysin-1 antibodies. Merged zoom image of synaptophysin-1 (green) and SNX4 (magenta). Scale bar of the neurons image = 20 μm, scale bar of the zoomed neurite = 5 μm. (**d**) Confocal microscopy images of neurons infected with control shRNA, the three shRNAs against SNX4 and corresponding rescue constructs. Left, mCherry signal reporting the transfection of the shRNAs; Middle, synaptophysin-1 labelling; Right, SNX4 labelling (n = 50 ± 13 fields of view, N = 4 ± 1 cultures. Scale bar = 20 μm. (**e**) Quantification of SNX4 labeling intensity relative to control. (**f**) Quantification of synaptophysin-1 staining intensity relative to control. Detailed information (average, SEM, n and statistics) is shown in Supplementary Table S1.

Commercially available antibodies against SNX4 only detect mouse SNX4 by western blot (Supplementary Fig. S1). In order to characterize the subcellular localization of endogenous SNX4 in mouse neurons, we developed a novel antibody. This antibody was designed in collaboration with Synaptic Systems (Cat. No. 392 003) against the N-terminal region of mouse SNX4 (Supplementary Fig. S1). To confirm that the antibody specifically detects SNX4, we developed three independent short hairpin RNAs (shRNAs) against SNX4 and rescue constructs in lentivirus. Cortical mouse neurons were infected with rescue SNX4 constructs at DIV3 (R1, R2 and R3) and at DIV7 with three shRNAs against SNX4 (shSNX4-1, shSNX4-2, and shSNX4-3) or an shRNA control (Control). At DIV14-15, the neurons were lysed or fixed and SNX4 levels were evaluated using western blot or immunocytochemistry (Fig. 1). On western blot, the antibody detected a protein of ~ 50 kDa, which corresponds with the size of SNX4. The intensity of this band was decreased by all three shRNAs against SNX4 and these levels were restored when shRNA was combined with the corresponding SNX4 rescue construct (Fig. 1a,b). SNX4 reduction was also verified by mass spectrometry (Supplementary Table S2, Supplementary Fig. S10). The same ~ 50 kDa band was observed using two commercially available antibodies (Supplementary Fig. S1). Our new antibody also detected a lower band of ~ 30 kDa in all samples that remained unaffected in neurons expressing shRNA against SNX4, suggesting that this antibody also binds an unspecific protein (Supplementary Fig. S1). When probing homogenates derived from 7 distinct regions of the mouse brain, the ~ 50 kDa band appeared in all region samples tested (Supplementary Fig. S1), suggesting that SNX4 is ubiquitously expressed in the mouse brain.

To test the application of the new antibody in immunocytochemistry, DIV15 cortical neurons were labelled with antibodies against SNX4 and synaptophysin-1 (a synaptic protein used as control) (Fig. 1c,d). mCherry reported the transfection of all shRNA constructs. Immunolabeling with the SNX4 antibody showed a punctate pattern in murine primary neuron cultures (Fig. 1c). The total SNX4 signal intensity was decreased upon SNX4 knockdown and was restored when shRNAs were combined with their rescue constructs (Fig. 1d–f). On the other hand, the total synaptophysin-1 intensity remained unchanged upon modulation of SNX4 levels (Fig. 1d–f). This indicates that the antibody signal was specific for SNX4 in immunocytochemistry. Together, these data confirm that SNX4 is detected using the novel antibody both in western blot and immunocytochemistry.

### SNX4 is located on early and recycling endosomes in neurons

**Figure 2:**
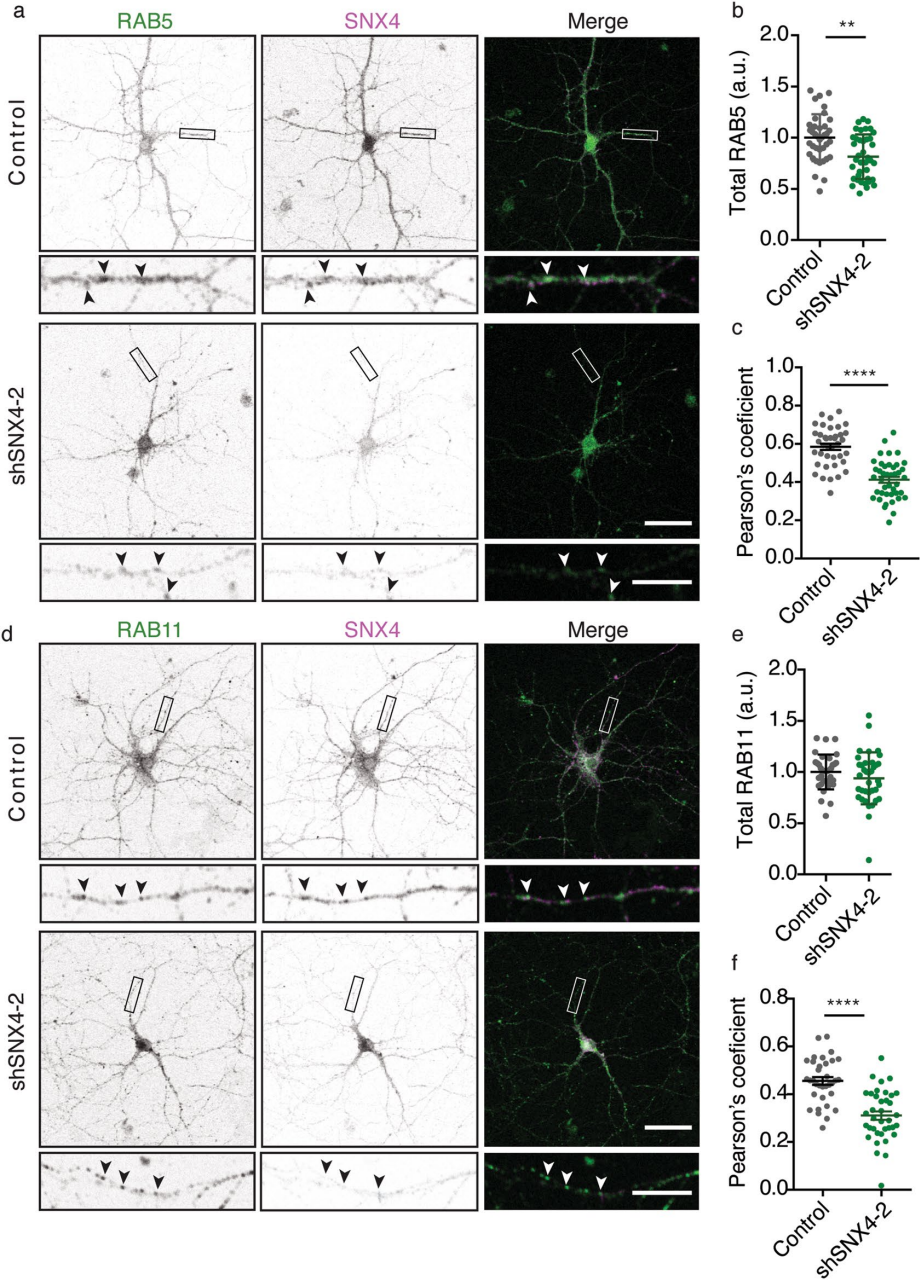
SNX4 is located on early and recycling endosomes in neurons. (**a**) Confocal microscopy images of control and SNX4 knockdown neurons labelled for RAB5 and SNX4. Merged image of RAB5 (green) and SNX4 (magenta). (n = 21 ± 2 neurons, N = 3 cultures). Scale bar of the neuron image = 50 μm, scale bar of the zoomed neurite = 5 μm. (**b**) Quantification of total neuronal RAB5 levels normalized to control. (**c**) Pearson’s coefficients for the co-localization of RAB5 and SNX4 in neurites. (**d**) Confocal microscopy images of control and SNX4 knockdown neurons labelled for RAB11 and SNX4. Merged image of RAB11 (green) and SNX4 (magenta). (n = 38 ± 1 neurons, N = 3 cultures). Scale bar of the neuron image = 50 μm, scale bar of the zoomed neurite = 5 μm. (**e**) Quantification of total neuronal RAB11 levels normalized to control. (**f**) Pearson’s coefficients for the co-localization of RAB11 and SNX4 in neurites. Detailed information (average, SEM, n and statistics) is shown in Supplementary Table S1.

SNX4 colocalizes with early and recycling endosome markers in HeLa cells, where it coordinates recycling from early endosomes to the plasma membrane through recycling endosomes^9^. To test if SNX4 also colocalizes with these endosome markers in neurons, hippocampal mouse neurons at DIV14-15 were fixed and labelled for endogenous SNX4 and RAB5 (early endosome marker) or RAB11 (recycling endosome marker). The specificity of SNX4 localization to these endosome markers was assessed by including SNX4 knockdown (shSNX4-2). SNX4 colocalized with RAB5 signal (Pearson's coefficient 0.58), which dropped significantly upon SNX4 depletion (Pearson’s coefficient 0.41, Fig. 2a,c). The total neuronal levels of RAB5 were 19% decreased upon SNX4 knockdown (Fig. 2b), corresponding to the smaller size of RAB5 puncta (Supplementary Fig. S6). Expression of shSNX4-2 did not affect the total neuronal levels of RAB11 (Fig. 2d,e). The Pearson’s coefficient for the colocalization between RAB11 signal and SNX4 signal was 0.45, which dropped to 0.31 upon SNX4 depletion (Fig. 2d,f). These data indicate that endogenous SNX4 localizes to early and recycling endosomes in cultured primary mouse neurons.

### SNX4 localizes to synaptic terminals

**Figure 3:**
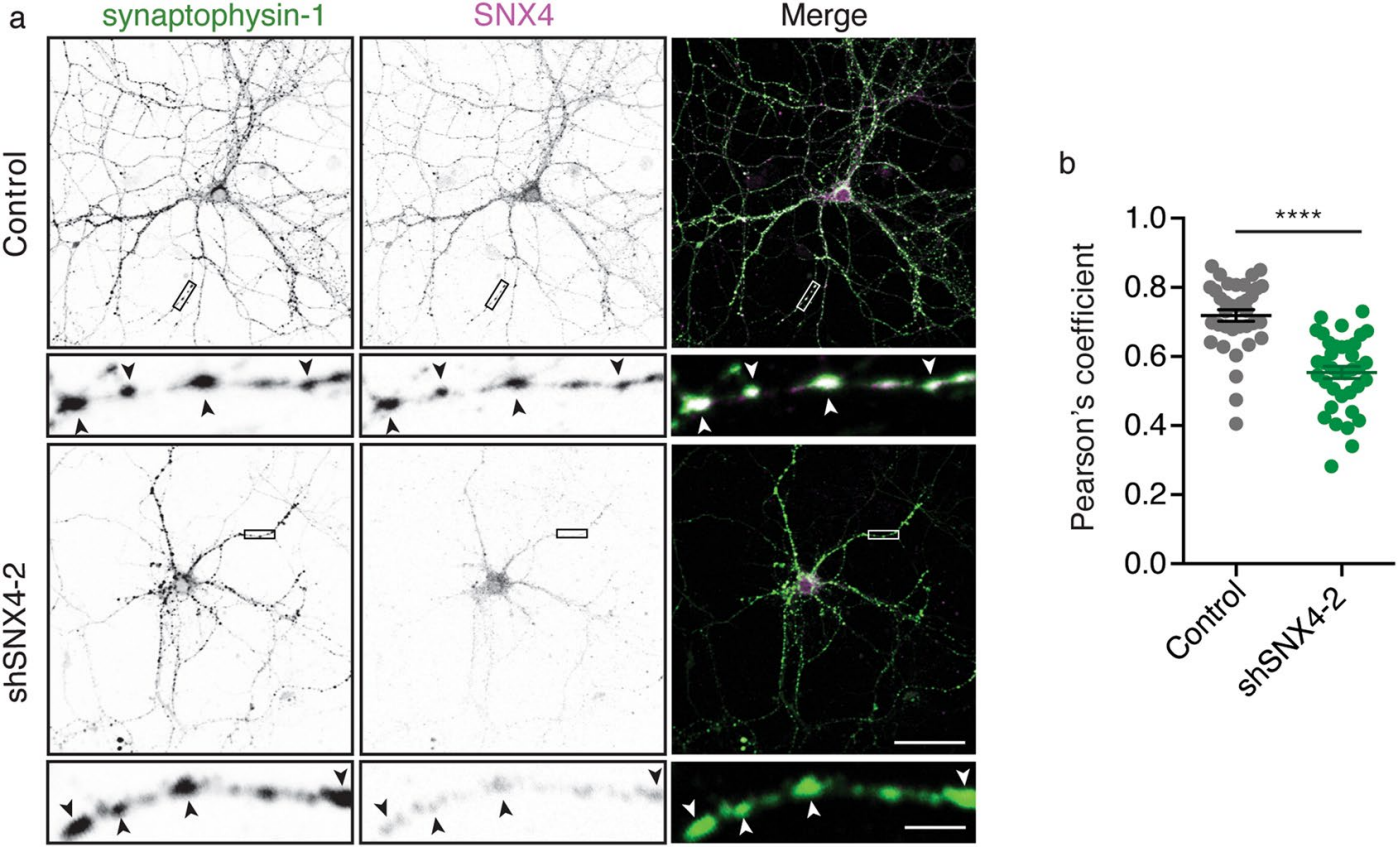
SNX4 is located to synapses. (**a**) Confocal microscopy images of control and SNX4 knockdown neurons labelled for synaptophysin-1 and SNX4. Merged image of synaptophysin-1 (green) and SNX4 (magenta). Scale bar of the neuron image = 50 μm, scale bar of the zoomed neurite = 5 μm. (**b**) Pearson's coefficient for the co-localization between synaptophysin-1 and SNX4 in neurites. (n = 36 fields of view, N = 3 cultures). Detailed information (average, SEM, n and statistics) is shown in Supplementary Table S1.

Immunoreactivity for SNX4 signal appeared to be present in synapses (Fig. 1c). To confirm this, we analysed the colocalization between SNX4 and synaptic markers. Control and SNX4 knockdown hippocampal neurons at DIV14-15 were fixed and labelled for SNX4 and synaptophysin-1(Fig. 3a). The Pearson’s coefficient for the colocalization between synaptophysin-1 signal and SNX4 signal was 0.71 (Fig. 3b). This value dropped to 0.55 upon SNX4 depletion (Fig. 3b). Similar effects were observed with the other two shRNAs against SNX4. The signal of SNX4 in synaptophysin-1 punctae was restored when the shRNAs were combined with their rescue constructs (Supplementary Fig. S3). The localization of endogenous SNX4 to synaptic structures was confirmed using bassoon, a marker of the presynaptic active zone (Supplementary Fig. S3). Taken together, these data demonstrate that SNX4 is targeted to synaptic locations.

**Figure 4:**
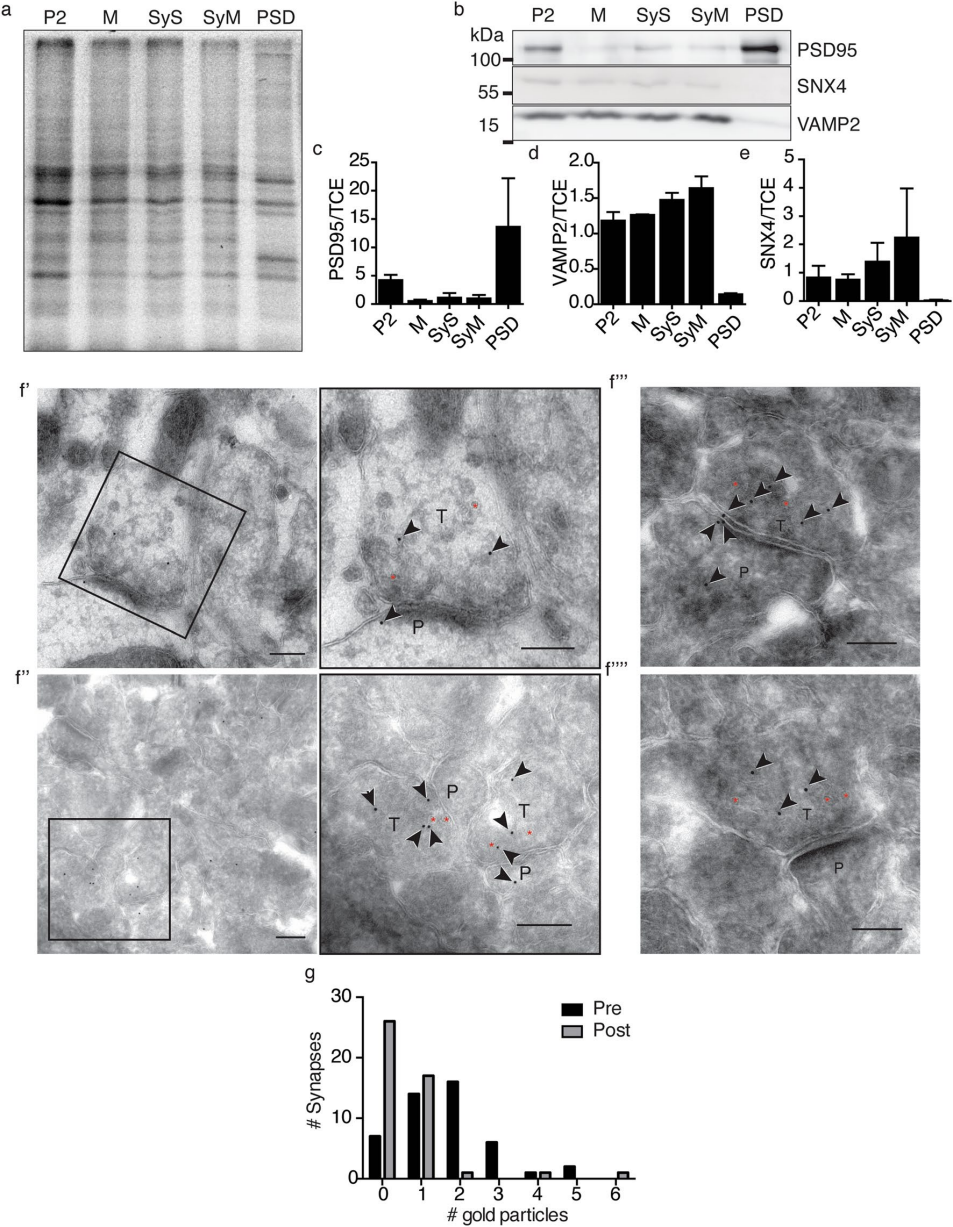
Synaptic SNX4 predominantly localizes to presynaptic terminals. (**a**) Total protein (TCE) in each hippocampal subcellular fraction (pellet 2 (P2), microsomal fraction (M), synaptosomes (SyS), synaptic membrane fraction (SyM), and PSD fraction (PSD)). (**b**) Representative cropped western blot of hippocampal subcellular fractions stained with SNX4, VAMP2/synaptobrevin-2 and PSD95. Original uncropped blots are shown in Supplementary Fig. S5. Quantification of (**c**) PSD95, (**d**) VAMP2/synaptobrevin-2 and (**e**) SNX4 levels normalized to total protein. Values are presented as a ratio compared to each total hippocampus lysates. (N = 3 blots/cultures). (**f**′, **f**′′, **f**′′′, **f**′′′′) Immuno-electron micrographs of synapses labelled for SNX4 and Protein A-10 nm gold conjugate. The images are representative of 3 independent experiments (N = 3 cultures). Scale bar = 200 nm. ‘P’ indicates postsynaptic side, ‘T’ the presynaptic terminal and ‘*’ is placed in the inside of some synaptic vesicles. (**g**) Number of gold particles at the postsynaptic side and the presynaptic terminal in each synapse (n = 46 synapses, N = 3 cultures). Detailed information (average, SEM, n and statistics) is shown in Supplementary Table S1.

To investigate the distribution of SNX4 within the synapse, we blotted subcellular fractions of the mouse hippocampus for SNX4 (Fig. 4a–e). SNX4 was detected in the synaptosome (SyS) fraction, corroborating a synaptic localization of SNX4. SNX4 was also present in the synaptic membrane fraction (SyM), but was not enriched in the PSD fraction (PSD). As expected, the PSD fraction was highly enriched in PSD95 and depleted of VAMP2/synaptobrevin-2. Therefore, the detection of SNX4 in hippocampal subcellular fractions is very similar to presynaptic markers such as VAMP2/synaptobrevin-2. To verify localization of SNX4 to specific synaptic compartments, immuno-gold electron microscopy was performed using Protein A-gold 10 nm to detect the SNX4 antibody. The gold particles were observed inside presynaptic terminals and on the postsynaptic membrane in close proximity to the postsynaptic density, but not within the postsynaptic density (Fig. 4f′,f′′,f′′′,f′′′′), in agreement with low SNX4 detection in the PSD fraction. Quantitative analysis showed that the SNX4 immunosignal was more abundant in the presynaptic than postsynaptic terminal (Fig. 4g). Little synaptic signal was detected in the negative controls (blocking peptide, Supplementary Fig. S4). Overall, these data show that SNX4 is present at both sides of the synapse, but is more abundant in presynaptic terminals.

### SNX4 depletion does not alter neuronal TNFR nor synaptic RAB11 levels

**Figure 5:**
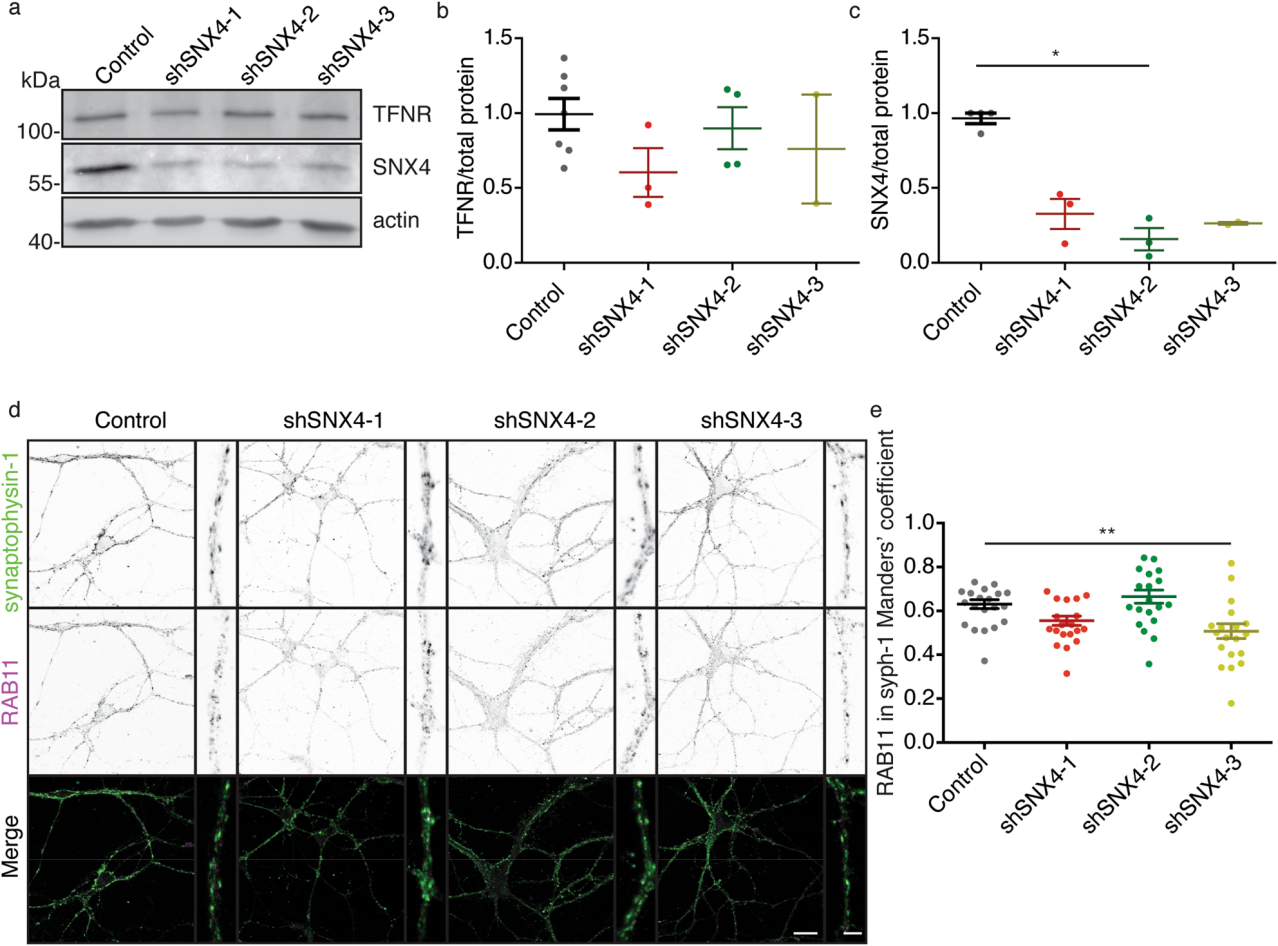
SNX4 depletion does not decrease TFNR levels in neurons nor recycling endosomal marker RAB11 levels at synapses. (**a**) Cropped western blot of neurons infected with control shRNA (Control) and the three shRNAs against SNX4 stained for TFNR, SNX4 and actin. Original uncropped blots are shown in Supplementary Fig. S5. (**b**) Quantification of TFNR levels normalized to total amount of protein (N = 3 ± 1). (**c**) Quantification of SNX4 levels normalized to total amount of protein in western blot (N = 3 ± 1). (**d**) Confocal microscopy images of control and SNX4 knockdown neurons labelled with synaptophysin-1 and RAB11 antibodies. Merged image of synaptophysin-1 (green) and RAB11 (magenta). (n = 21 ± 1 neurons, N = 2 cultures). Scale bar of the neuron image = 20 μm, scale bar of the zoomed neurite = 4 μm. (**e**) Manders’ coefficient for the co-localization of synaptophysin-1 and RAB11. Detailed information (average, SEM, n and statistics) is shown in Supplementary Table S1.

In non-neuronal cells, SNX4 depletion leads to an abnormal RAB11 distribution (from juxtanuclear to peripheral localization) and missorting and degradation of TFNR that is recycled by RAB11-positive endosomes^9^. To test if this SNX4 recycling pathway is also critical to prevent TFNR degradation in neurons, we measured the levels of TFNR upon SNX4 knockdown by western blot. Upon shSNX4 expression, TFNR levels were not changed by western blot (Fig. 5a–c, Supplementary Fig. S5) and by mass spectrometry (Control = 1.00 ± 0.08, shSNX4-1 = 0.47 ± 0.05, shSNX4-2 = 1.39 ± 0.07 and 1.14 ± 0.07 a.u.) (see Supplementary Table S2 for details). As reported above, the total level of RAB11 was not affected by SNX4 knockdown (Fig. 2e). However, the density of RAB11 endosomes in neurites was reduced while their size was increased by shSNX4-2 expression (Supplementary Fig. S6c,d). Upon SNX4 depletion, no difference was observed in the colocalization of the synaptic marker synaptophysin-1 with the recycling endosome marker RAB11 nor in the intensity of RAB11 signal within the synaptophysin-1 punctae (Fig. 5d,e, Supplementary Fig. S6). Also, the synaptic density (number of synapses per µm of neurite) was not affected in neurons expressing shRNA against SNX4 (Supplementary Fig. S7). These data indicate that the impact of SNX4 depletion on TFNR recycling and recycling endosomes is different in neurons compared to non-neuronal cells.

### The neuronal proteome is dysregulated upon SNX4 knockdown

**Figure 6:**
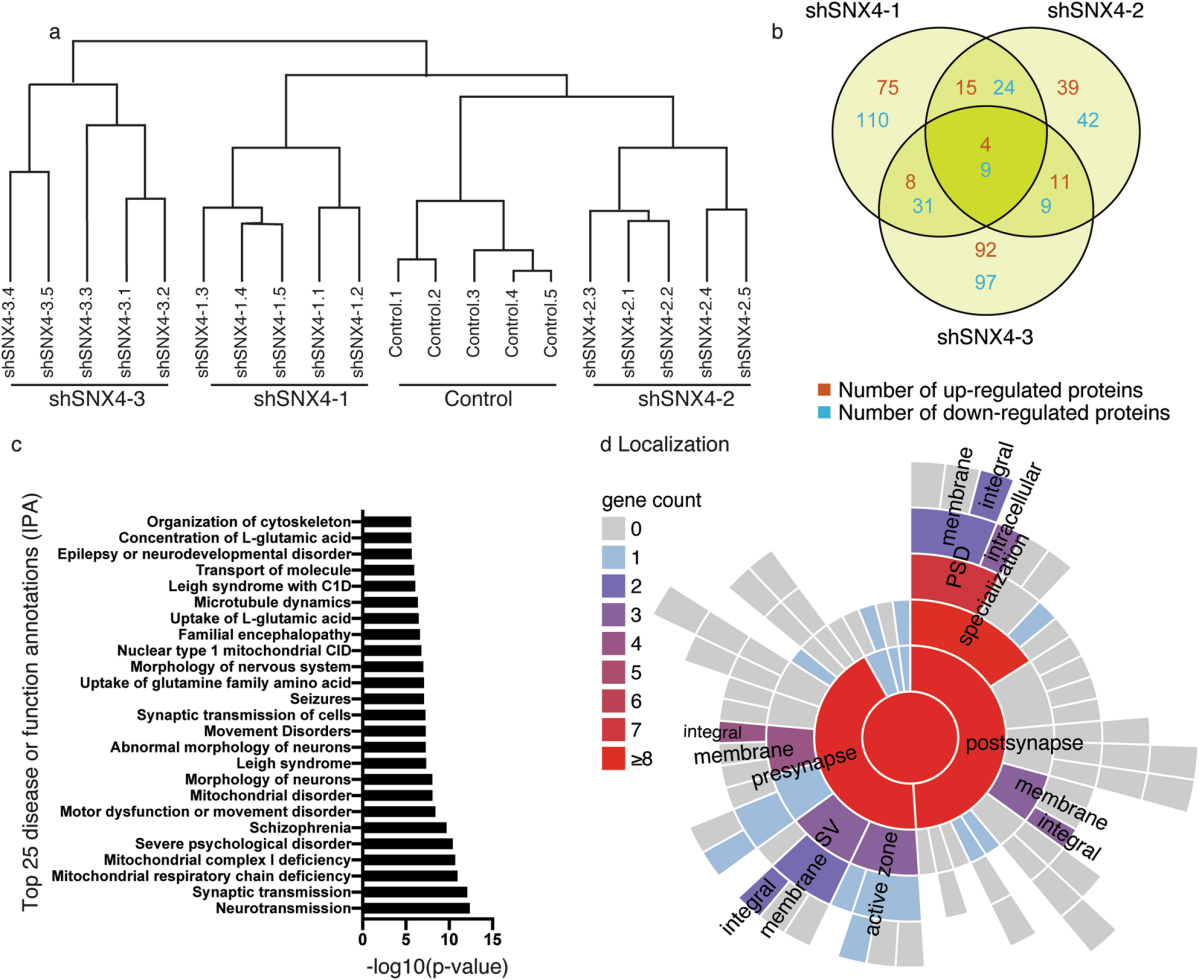
SNX4 depletion dysregulates the neuronal proteome. (**a**) Dendrogram of the protein expression relationship between neurons containing shRNAs against SNX4 or control shRNA. The hierarchical clustering reflects similarity between the samples. (**b**) Venn diagram showing the overlap among the dysregulated proteins in neurons containing shRNA against SNX4 compared with control. (**c**) Functional analysis of the differentially expressed proteins in the same direction in at least two of the SNX4 shRNA-expressing neurons revealing the 25 most enriched proteins groups. (**d)** Sunburst plot showing the annotation in synaptic location of the altered proteins in the same direction in at least two shRNAs against SNX4 groups using SynGO^15^ analysis. (Output plot of SynGO 1.0 dataset version/release: 20180731). Inner rings are parent terms of more specific child terms in the outer rings, colour coded according to gene count in each term.

To explore the function of neuronal SNX4, the proteome of SNX4 knockdown and control neurons was compared. Five independent cortical neuron cultures were infected with control shRNA and three different shRNAs against SNX4 at DIV7. At DIV15, cells were harvested and the proteins were extracted and digested into peptides for subsequent identification and quantification using LC–MS/MS^13,14^. A total of 2531 proteins were identified and quantified from 12,027 peptides. Only peptides identified with high confidence were used (i.e., a Q-value ≤ 0.01 over all samples in at least one group, allowing for one outlier within each condition). The full list of proteins quantified in this study is represented in Supplementary Table S2. Hierarchical clustering was used to classify samples into groups according to similarity. Biological replicates from the same experimental group (control, shSNX4-1, shSNX4-2 and shSNX4-3) clustered together (Fig. 6a). This indicates that the expression of each individual SNX4-targetted shRNA results in a distinct and reproducible neuronal proteome. Compared with control, 290, 167 and 283 proteins were dysregulated in shSNX4-1, shSNX4-2 and shSNX4-3 expressing neurons, respectively (Supplementary Fig. S8). Among the 134 significantly regulated proteins in at least 2 shRNAs, 4 were found up-regulated and 9 down-regulated in all knockdown groups (Fig. 6b). Although fold change was not a cut off criteria, a 20% or higher degree of change was observed among the proteins significantly dysregulated in the same direction in the three shRNAs against SNX4 (the smaller change observed was a 24.55% reduction in NSF equivalent to -0.463 log2, Supplementary Fig. S9).

To further study the effect of SNX4 depletion on the neuronal proteome in an unbiased manner, we interrogated the 111 proteins significantly dysregulated in the same direction in at least two shRNAs using Ingenuity Pathway Analysis (IPA). The functional annotation indicated neurotransmission and synaptic transmission as the most significant overrepresentation. Moreover, disease annotations such as severe psychological disorders, schizophrenia and mitochondrial disorder were enriched. In addition, transport of molecules was among the 25 annotations with the lowest *p* values, in line with the established protein sorting role of SNX4 (Fig. 6c). All the information generated from the IPA analysis, including protein identities for each process, is shown in Supplementary Table S3.

We further characterized the synaptic location and functions of the individual synaptic proteins altered upon SNX4 depletion using SynGO^15^. From the 111 proteins significantly dysregulated in the same direction in at least two shRNAs, 30 were mapped to SynGO annotated genes which are based on expert-curated literature evidence^15^ (Supplementary Table S4). Twenty-nine genes have a Cellular Component annotation, many of which encoded for *membrane integral* proteins in different components of the *pre* and *postsynapse*, suggesting that SNX4 functions in the sorting of synaptic membrane proteins (Fig. 6d, Supplementary Table S4). Twenty-two genes were mapped for a wide variety of Biological Processes annotations which included *metabolism, synapses assembly* in *organization*, *chemical transmission* in *signaling*, neurotransmitter levels (*NTR levels*) in *postsynapti*c, *exocytosis* and *endocytosis* in *synaptic vesicle cycle* (*SV cycle*) in *presynaptic* (Supplementary Fig. S10, Supplementary Table S4), implicating SNX4 in a variety of synaptic processes.

Taken together, SNX4 knockdown regulated the expression of multiple proteins involved in neurotransmission and related diseases on both the pre- and postsynaptic side of the synapse. These experiments suggest that SNX4 plays a role in synaptic proteome regulation.

## Discussion

SNX4 localizes on early and recycling endosomes in mitotic cells^5,7,9,16,17^. Also, in neurons, we found that SNX4 co-localized with early (RAB5) and recycling (RAB11) endosome markers, suggesting that neuronal SNX4 functions in the previously described recycling pathway from early endosome to the plasma membrane through the recycling endosome. In HeLa cells, SNX4 silencing leads to abnormal early and recycling endosome distribution: A shift from juxtanuclear to peripheral distribution due to perturbation of early-to-recycling endosome transport^9^. Perhaps, a similar perturbation occurs in neurons, since expression of shSNX4-2 decreased total RAB5 intensity and puncta size and decreased the density of RAB11 punctae while increasing their size (Fig. 2b and Supplementary Fig. S6). The best described SNX4-dependent recycling cargo is TFNR, which is decreased upon SNX4 depletion in HeLa cells^9^. In neurons, SNX4 depletion did not decrease TFNR levels measured by western blot or mass spectrometry (Fig. 5, Supplementary Table S2). Therefore, the impact of SNX4 depletion on TFNR levels in neurons is different compared to HeLa cells, but this does not fully exclude that neuronal TFNR is recycled by SNX4. For example, SNX4-dependent cargo might not be degraded in lysosomes upon SNX4 depletion in neurons but instead mislocalize or accumulate in internal compartments without affecting the total protein level. Nevertheless, SNX4-dependent recycling seems to be different in neurons, which may reflect a differential demand of this pathway in these large, polarized, post-mitotic cells specialized in neurotransmission.

In neurons, endogenous SNX4 highly colocalizes with synaptic markers (Fig. 3 and Supplementary Table S1) and immuno-electron microscopy revealed that endogenous SNX4 is present in both pre- and postsynaptic terminals. In agreement with this synaptic localization, explorative mass spectrometry showed that SNX4-targetted shRNA expression results in dysregulation of the neuronal proteome, including proteins involved in neurotransmission and associated with severe psychological disorders (Fig. 6c). Many of the synaptic proteins dysregulated by SNX4 knockdown were mapped as integral membrane proteins at both sides of the synaptic terminal (Fig. 6d). Given the established role of SNX4 in endosomal sorting of transmembrane proteins^8,9^, we speculate that SNX4 is involved in endosomal sorting of synaptic membrane proteins. On the postsynaptic side, the endosomal system is involved in the insertion of integral neurotransmitter receptors in the plasma membrane, which is a mechanism of synaptic plasticity (see review^18^). For example, depletion of the endosomal sorting protein VPS35 leads to decreased levels of glutamate receptors in the plasma membrane, reducing glutamatergic neurotransmission^19–21^. The glutamate ionotropic receptor AMPA type subunit 1 (gene GRIA1, Supplementary Table S2, Supplementary Fig. S9) is downregulated by SNX4 knockdown. This suggests that SNX4 may function in the same or a similar sorting pathway of glutamate receptors. In presynaptic terminals, the protein levels of calcium sensors such as the synaptic vesicle component synaptotagmin-1 and the calcium dependent secretion activator CAPS1 are dysregulated upon SNX4-targetted shRNA expression (Supplementary Table S2, Supplementary Fig. S9). This suggests that SNX4 may sort these membrane proteins in the presynaptic terminal to regulate calcium-dependent synaptic vesicle exocytosis. Furthermore, increased lysosomal degradation of SNX4-dependent cargo has been described when SNX4 is depleted in non-neuronal cells^9^. The expression of the lysosomal proteins Cathepsin D and LAMP1 was found increased upon SNX4 depletion in neurons, which suggest a higher demand for lysosomal degradation upon SNX4 knockdown (Supplementary Fig. S9). Uncovering synaptic SNX4 sorting pathways will provide insight into the homeostasis of the local synaptic proteome, on which these distant synaptic terminals may rely.

We found that SNX4 locates to synapses and that synaptic membrane proteins are dysregulated upon SNX4 knockdown in neurons. This suggests that SNX4-dependent sorting is functionally relevant for synapses. However, the precise role of SNX4 in neurons and in particular in synapses remains to be elucidated. Numerous hypotheses could emerge from our data. To enable this exploration, we provided the complete dataset in tabular format (Supplementary Table S2). The proteomic analysis of SNX4-depleted neurons shows that each individual shRNA induces a reproducible but distinct dysregulation of the neuronal proteome (Fig. 6), which is difficult to explain by the depletion of SNX4 alone. Likely, each shRNA against SNX4 affects a distinct SNX4-independent group of proteins, these so-called off-target effects are well described for shRNA technology (see reviews^22,23^) Hence, validation of these hits, including a rescue experiment using shRNA-resistant constructs to assure that the effect depends on the target SNX4, is recommended before further functional studies are undertaken. Notwithstanding these limitations, the localization of SNX4 at synaptic terminals opens new avenues for research into the role of this protein at synapses, which may provide a better understanding of the local regulation of the synaptic proteome.

## Materials and methods

### Plasmids

shRNAs were cloned into a lentiviral expression vector under the U6 promotor. To report lentiviral infection, the plasmid also contained mCherry under the synapsin promotor. The target sequences of the shRNAs were as follows: GGG AAT GAC TAC CAA ACT C (shSNX4-1), GCA GTG GAA TAG ATA CAT TAT (shSNX4-2), GCT GAT ATT GAA CGC TTC AAA (shSNX4-3), TTC TCC GAA CGT GTC ACG T (Control, scramble)^24^. Mouse SNX4 cDNA was used to induce the silent point mutations in rescue constructs. The following forward primers were used: GAAGGGAATGACAACGAAGCTTTTTGGTCAAGAAACTCCAG (shSNX4-1) GGGCTGATATCGAGCGCTTTAAAGAACAAAAG (shSNX4-3).

### Laboratory animals

Animal experiments were approved by the animal ethical committee of the VU University/VU University Medical Centre (“Dier ethische commissie (DEC)”; license number: FGA 11-03) and are in accordance with institutional and Dutch governmental guidelines and regulations.

### Primary cell culture

Primary neurons were cultured from mouse E18 hippocampi or cortices. Briefly, tissue was dissected in Hanks balance salt solution (HBSS, Sigma) with 10 mM HEPES (Life Technologies) and digested by 0.25% trypsin (20 min at 37 °C; Life technologies) in HBSS. The tissue disociation was performed with fire-polished Pasteur pipettes in DMEM with FCS. The neurons were spun down and re-suspended in neurobasal medium with 2% B-27, 18 mM HEPES, 0.25% glutamax and 0.1% Pen-Strep (Life Technologies). Neurons were plated on coverslips pre-coated with poly-L-ornithine (Sigma) and laminin (Sigma) or on an astrocyte monolayer. Neurons were maintained at 37 °C and 5% CO_2_ until the day of the experiment.

### Subcellular fractioning

Subcellular fractions were obtained from hippocampi from three-month-old C57BL6 mice as previously described^25,26^. Isolated hippocampi were homogenized on a dounce homogenizer (potterS; 12 strokes, 900 rpm) using homogenizer buffer (0.32 M Sucrose, 5 mM HEPES pH 7.4, Protease inhibitor cocktail (Roche)), and spun at 1000×*g* for 10 min at 4 °C to obtain Supernatant 1 (S1). S1 was centrifuged at 20,000×*g* for 20 min to obtain pellet 2 (P2) and supernatant 2 (S2). S2 was ultracentrifuged at 100,000×*g* for 2 h to obtain the pellet containing the microsomal fraction (M). S1 was ultracentrifuged in a 0.85/1.2 M sucrose density gradient at 100,000×*g* for 2 h to obtain Synaptosomes (SyS) at the interface of 0.85/1.2 M sucrose. SyS were exposed to a hypotonic shock of 5 mM HEPES pH 7.4 with protease inhibitor for 15 min, and sucrose gradient ultracentrifuged as stated above to obtain the synaptic membrane fraction (SyM) at the interface of 0.85/1.2 M. SyS was also treated with 1% Tx-100 for 30 min, layered on top of 1.2/1.5/2 M sucrose, centrifuged at 100,000×*g* for 2 h, to obtain the PSD fraction (PSD) at the interface of 1.5/2 M sucrose.

### Western blot

Wild type mouse brain regions were homogenized in ice-cold phosphate-buffered saline (PBS) with protease inhibitors and lysed (100 µl Laemmli sample buffer (2% w/v sodium dodecyl sulfate (SDS), 10% v/v Glycerol, 0.26 M β-mercaptoethanol, 60 mM Tris–HCl pH 6.8, and 0.01% w/v bromophenolblue) per each mg). DIV14-15 cortical neurons were washed with ice-cold PBS, scraped, lysed in Laemmli sample buffer. Samples were boiled for 10 min at 90 °C, loaded in SDS-PAGE (10% 1 mm acrylamide gel with 2,2,2-Trichloroethanol) and transferred into Polyvinylideenfluoride (PVDF) membranes (Bio-rad) (1 h, 0.3 mA, 4 °C). Membranes were blocked using 2% milk (Merck) with 0.05% of normal goat serum (NGS) in PBS-T (PBS with 0.1% Tween-20), incubated overnight at 4 °C with the primary antibodies in PBS-T (Supplementary Table S5), with secondary alkaline phosphatase-conjugated antibodies (1:10,000, Jackson ImmunoResearch) in PBS-T during 1 h at 4 °C and incubated 5 min with AttoPhos (Promega). Images were acquired with a FLA-5000 fluorescent image analyzer (Fujifilm) and analysed with ImageJ Gel Analysis.

### Immunocytochemistry and confocal imaging

Neurons at DIV 14–15 were fixed with 2% paraformaldehyde in PBS and cell culture medium for 10 min followed by 4% paraformaldehyde in PBS for 30 min at room temperature. Then, neurons were washed, permeabilized with 0.5% Triton X-100 for 5 min, blocked with 2% normal goat serum and 0.1% Triton X-100 in PBS for 40 min, incubated for 1 h with primary antibodies (Supplementary Table S5), for 1 h with secondary antibodies conjugated to Alexa dyes (1:1000, Molecular Probes) and mounted on microscope slides with Dabco-Mowiol at room temperature. Images were acquired using a Carl Zeiss LSM510 meta confocal microscope with a Plan-Neofluar 40x/1.3 oil objective and a Nikon Eclipse Ti with 63x/1.4 oil objective controlled by NisElements 4.30 software. Imaging acquisition settings and contrast were kept constant between the experimental conditions within each experiment. Colocalization was quantified Pearson’s coefficient which correlates the pixel intensity in both channels and Manders’ colocalization coefficient which assesses the co-occurrence of the signal of both channels using pixel by pixel comparison by the JACoP plugin^27,28^ For quantification of protein levels, intensity was measured inside a neuronal mask in ImageJ.

### Electron microscopy

Hippocampi of 2-month-old mice were fixed in 4% PFA with 0.1% glutaraldehyde in 0.1 M PB and embedded in increasing concentrations of gelatine at 37 °C (5 min 2% gelatine, 15 min 5% gelatine, 30 min 10% gelatine, 10 min 12% gelatine, 60 min 12% gelatine). The hippocampi were infiltrated in 2.3 M sucrose at 4 °C and frozen in liquid nitrogen. 70 nm thick sections were obtained with a cryo-ultramicrotome (UC6, Leica), collected at − 120 °C in 1% methylcellulose and 1.2 M sucrose and transferred onto formvar/carbon-coated copper mesh grids. The sections were washed with PBS at 37 °C, treated with 0.1% glycine, blocked with 0.1% of BSA and 0.1% cold water fish gelatine, incubated for 2 h with SNX4 antibody in blocking solution (Supplementary Table S5) and for 1 h with Protein A-10 nm gold (1: 25, CMC, UMC Utrecht, Netherlands) at room temperature. The sections were counterstained with 0.4% uranyl acetate in 1.8% methylcellulose on ice and imaged on a Tecnai 12 Biotwin transmission electron microscope (FEI company). Synapses were recognized by the presence of ~ 40 nm-sized vesicles in the presynaptic compartment opposed by a densely stained membrane region on the postsynaptic compartment, indicative of a postsynaptic density.

### Proteomics

DIV14-15 cortical neurons (500.000 neurons per sample) were prepared as previously described^29^. In brief, cells were washed and collected in ice cold PBS with protease inhibitor cocktail (Roche). Neurons were pelleted (5 min at 3000 g at 4 °C), lysed in Laemmli loading buffer, boiled at 90 °C for 5 min and incubated with acrylamide for 30 min. In-gel digestion with Trypsin/Lys-C Mix solution (Promega) was performed overnight at 37 °C. The peptides were dried using a speedvac and stored at − 20 °C.

The peptides were re-dissolved in 2% acetonitrile/0.1% formic acid solution containing iRT reference peptides and injected into the Ultimate 3000 LC system. The peptides were trapped on a 5 mm C18 PepMap 100 column for 5 min and separated on a homemade 200 mm C18 Alltima column. The reverse phase liquid chromatography was performed by linearly increasing the acetonitrile concentration in the mobile phase at a flow rate of 5 μL/min: from 5 to 22% in 88 min, to 25% at 98 min, to 40% at 108 min and to 95% in 2 min. The separated peptides were electro-sprayed into the TripleTOF 5600 MS (Sciex) with a micro-spray needle (at a voltage of 5500 V). The mass spectrometer was set in data-independent acquisition at high sensitivity and positive mode under the following parameters: parent ion scan of 100 ms (mass range of 350–1250 Da), SWATH mass range between 450–770 m/z, SWATH window of 8 Da, MS/MS scan time of 80 ms per window (range 200–1800 Da), collision energy for each window was determine for a 2 + ion centered upon the window, with a spread of 15 eV.

Data was analysed using Spectronaut 8.0^30^ and a spectral library created from merging two data-dependent analyses of wild type hippocampal neuron cultures and hippocampal synaptosomes containing spike-in iRT peptides from Biognosys^31^. The retention time prediction was set to dynamic iRT; the cross-run normalization based on total peak areas was enabled. Protein abundances were computed using Spectronaut normalized peak area, and Loess normalized using the ‘normalizeCyclicLoess’ function from limma R package (fast method and 10 iterations)^32^. Proteins significantly dysregulated in the same direction in at least two shRNA against SNX4 expressing groups were imported into IPA for core analysis without user intervention. Direct and indirect relationships were considered for the analysis of all data sources, mutation and species available with experimentally observed confidence level. Finally, these proteins were annotated using SynGO^15^.

### Statistical analysis

Data are expressed as mean values ± standard error of the mean (SEM). The Shapiro–Wilk normality test was used to evaluate the distribution of the data. Bartlett’s test was used to test homoscedasticity. When comparing two homoscedastic and normal distributed groups, t-test was used. Other two-group comparisons were analysed by the Mann–Whitney test. When comparing more than two groups and the data were normally distributed and homoscedastic, a one-way analysis of variance (ANOVA) was used. Dunnett’s post-hoc tests were performed after a significant effect was detected by comparing the different knockdown groups to the control. In case of comparing more than two groups and the data were not normality distributed and homoscedastic, the Kruskal–Wallis test was used with Dunn’s multiple comparison test as post-hoc. For proteomic analysis, empirical Bayes moderated t-statistics with multiple testing correction by false discovery rate (FDR) was performed on log-transformed protein abundances as implemented by the ‘eBayes’ and ‘topTable’ functions from limma R package. Proteins were considered significantly regulated with a FDR corrected *p* value ≤ 0.01. When *p* values were lower than 0.05, significance was noted in the figure as: *P < 0.05, **P < 0.01, ***P < 0.001, ****P < 0.0001.

## Supplementary information

Supplementary Information.

Supplementary Tables.

## Data Availability

The datasets generated and analysed during the current study are available from the corresponding author on request.
