# Supplementary material for: The endosomal protein sorting nexin 4 is a synaptic protein
Source: Sci Rep. 2020 Oct 26;10:18239. doi: 10.1038/s41598-020-74694-6 (PMC7588491; doi:10.1038/s41598-020-74694-6)
Supplement: Supplementary file 1 — Supplementary Information. [file 41598_2020_74694_MOESM1_ESM.pdf]

Supplementary information

The endosomal protein sorting nexin 4 is a synaptic protein

Sonia Vazquez-Sanchez[1], Miguel A. Gonzalez-Lozano[2], Alexarae Walfenzao[1], Ka Wan Li[2], and Jan R.T. van Weering [3, *]

[1]*Department of Functional Genomics, Center for Neurogenomics and Cognitive Research, Amsterdam Neuroscience, VU University, Amsterdam, The Netherlands*

[2]*Department of Molecular and Cellular Neurobiology, Center for Neurogenomics and Cognitive Research, Amsterdam Neuroscience, VU University, Amsterdam, The Netherlands*

[3]*Clinical Genetics, Center for Neurogenomics and Cognitive Research, Amsterdam Neuroscience, Amsterdam UMC location VUmc, The Netherlands*

[*]**Corresponding author:** Jan R.T. van Weering, Clinical Genetics, Center for Neurogenomics and Cognitive Research, Amsterdam UMC location VUmc, De Boelelaan 1085, 1081 HV Amsterdam, The Netherlands.
Email: jan.van.weering@vu.nl

a

```
MEQAPPDPEKLLQPGPLEPLGGPGAVLEAAVGEENEGTREDGSGVDTMTGNNFWLKKIEISVSEAEKRTG
RNAVNMQETYTAYLIETRSVEHADGQSVLTDSLWRRYSEFELLRNYLLVYYPHVVVPPLPEKRAEFVWHK
LSADNMDPDFVERRRVGLENFLLRVASHPVLCRDKIFYSFLTQEGNWKETVNETGFQLKADSRLKALNAT
FRVKNPDKRFTELRHYSDELQSVISHLLRVRARVADRLYGVYKVHGNYGRVFSEWSAIEKEMGDGLQSAG
HHMDVYASSIDDILEDEEHYADQLKEYLFYAEALRAVCRKHELMQYDLETAAQDLAAKKQQCEELATGTV
RTFSLKGMTTKLFGQETPEQREARIKVLEEQINEGEQQLKSKNLEGREFVKNAWADIERFKEQKNRDLKE
ALISYAVMQISMCKKGIQVWTNAKECFSKM
```

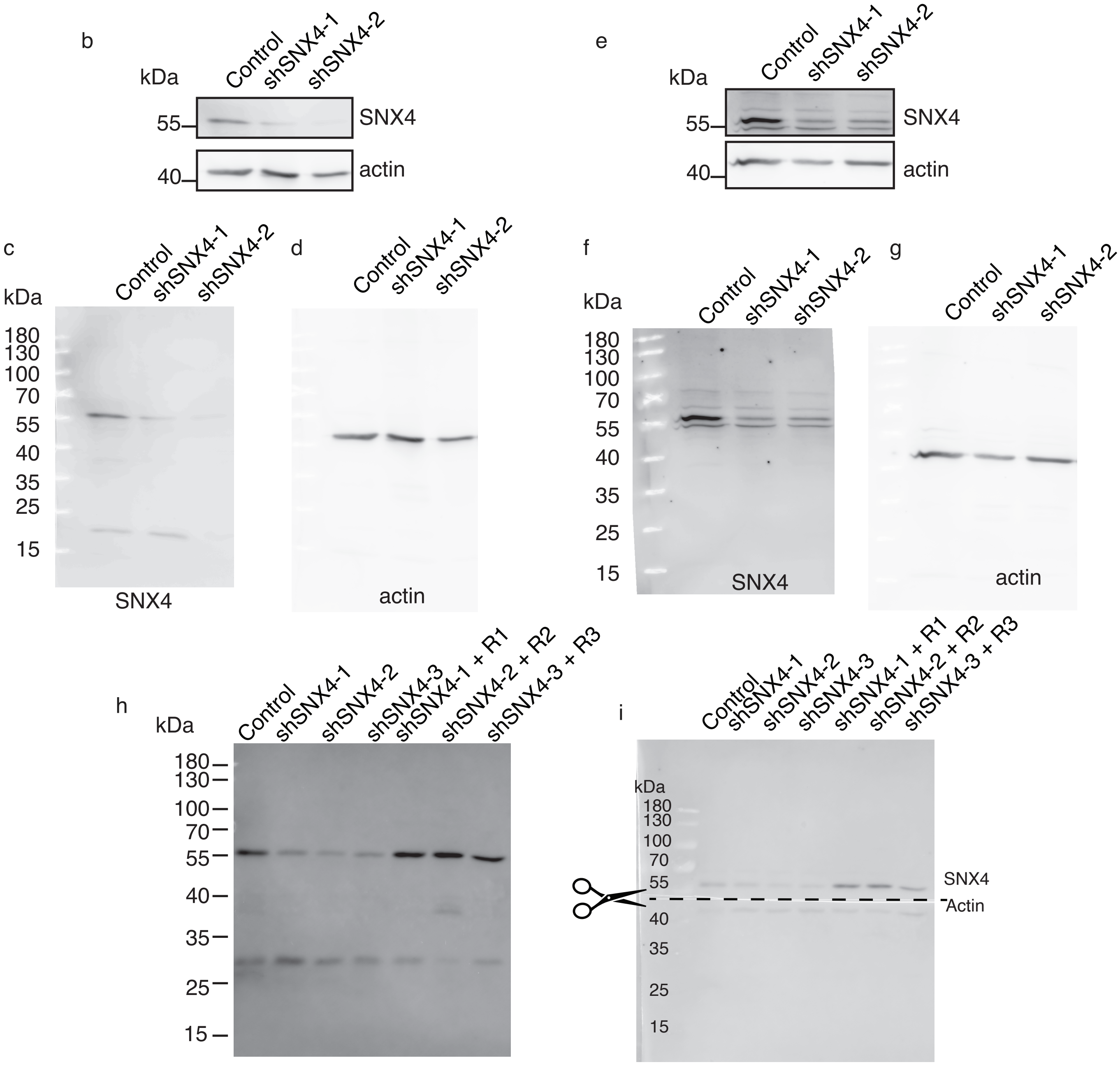

Supplementary Figure S1: Epitopes of the different antibodies against SNX4 (a) Sequence of amino acids of mouse SNX4 (>gi|18017596|ref|NP_542124.1| sorting nexin-4 [Mus musculus]). The epitopes of the different antibodies are highlighted. In orange, the epitope of SNX4 antibody from cat. N. 392 003, Synaptic Systems (1-21 amino acids of mouse SNX4). In blue, epitope from cat. N. HPA005709, Sigma (238-386 amino acids of human SNX4). In yellow, epitope from cat. N. sc-271403, Santa Cruz (361-393 amino acids of human SNX4). The green is just the product of the overlapping sequences highlighted in yellow and blue. (b) Representative western blot of control neurons and neurons with shRNAs against SNX4 stained for SNX4 (N. sc-271403, Santa Cruz) and actin. Original uncropped blots for SNX4 (N. sc-271403, Santa Cruz) (c) and actin (d). (e) Representative western blot of control neurons and neurons with shRNAs against SNX4 stained for SNX4 (N. HPA005709, S) and actin. Original uncropped blots for SNX4 (N. HPA005709, S) (f) and actin (g). (h) Full blot for SNX4 shown in Figure 1a. (i) Original uncropped blot from Figure 1a. The membrane was cut as indicated by 'scissors and dash line' and re-incubated with actin antiboy after SNX4 antibody incubation.

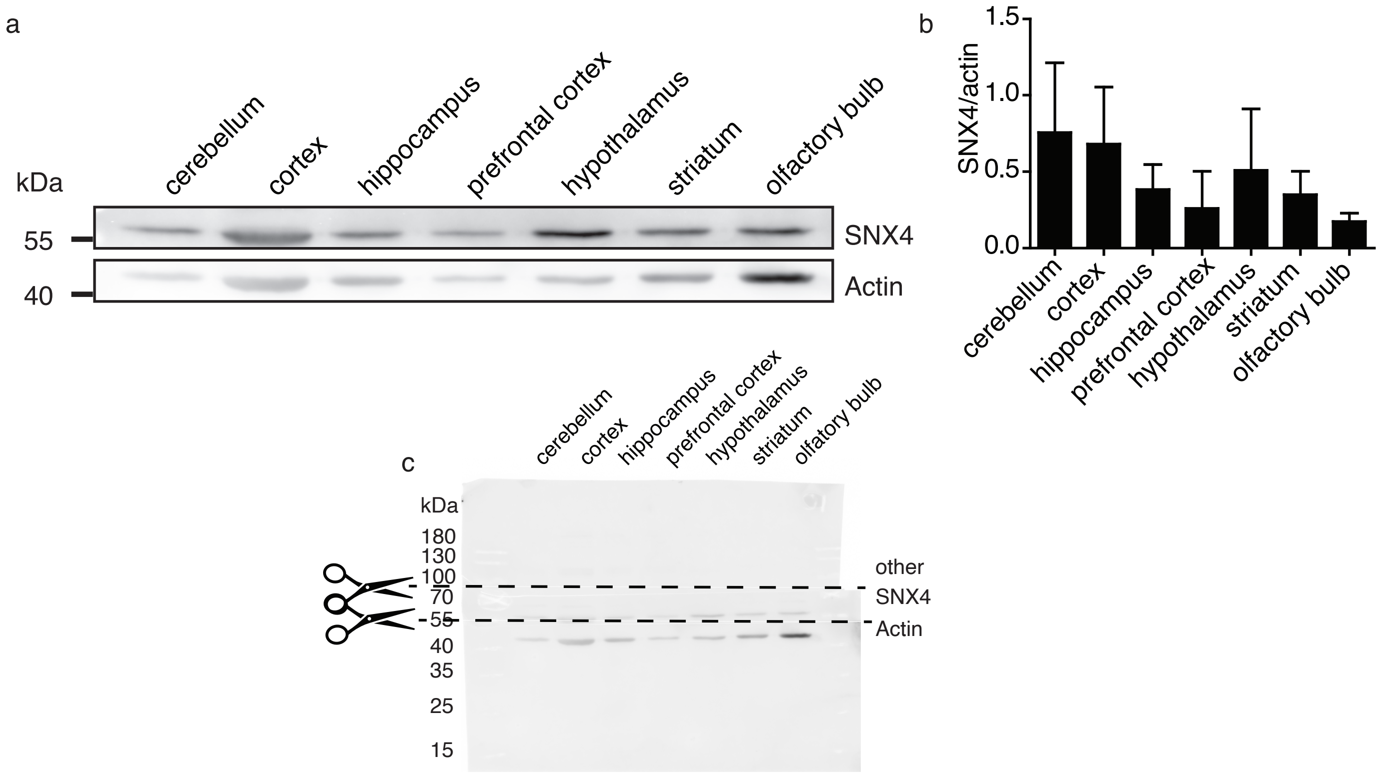

Supplementary Figure S2: SNX4 is expressed in the brain and in neurons. (a) Western blot of different mouse brain areas for SNX4 and actin. (b). Quantification of SNX4 levels normalized to actin in western blot. (N=2 or 3 blots/cultures). (c) Original uncropped blot. The membrane was cut as indicated by 'scissors and dash line' and each piece was incubated with the indicated primary antibody.

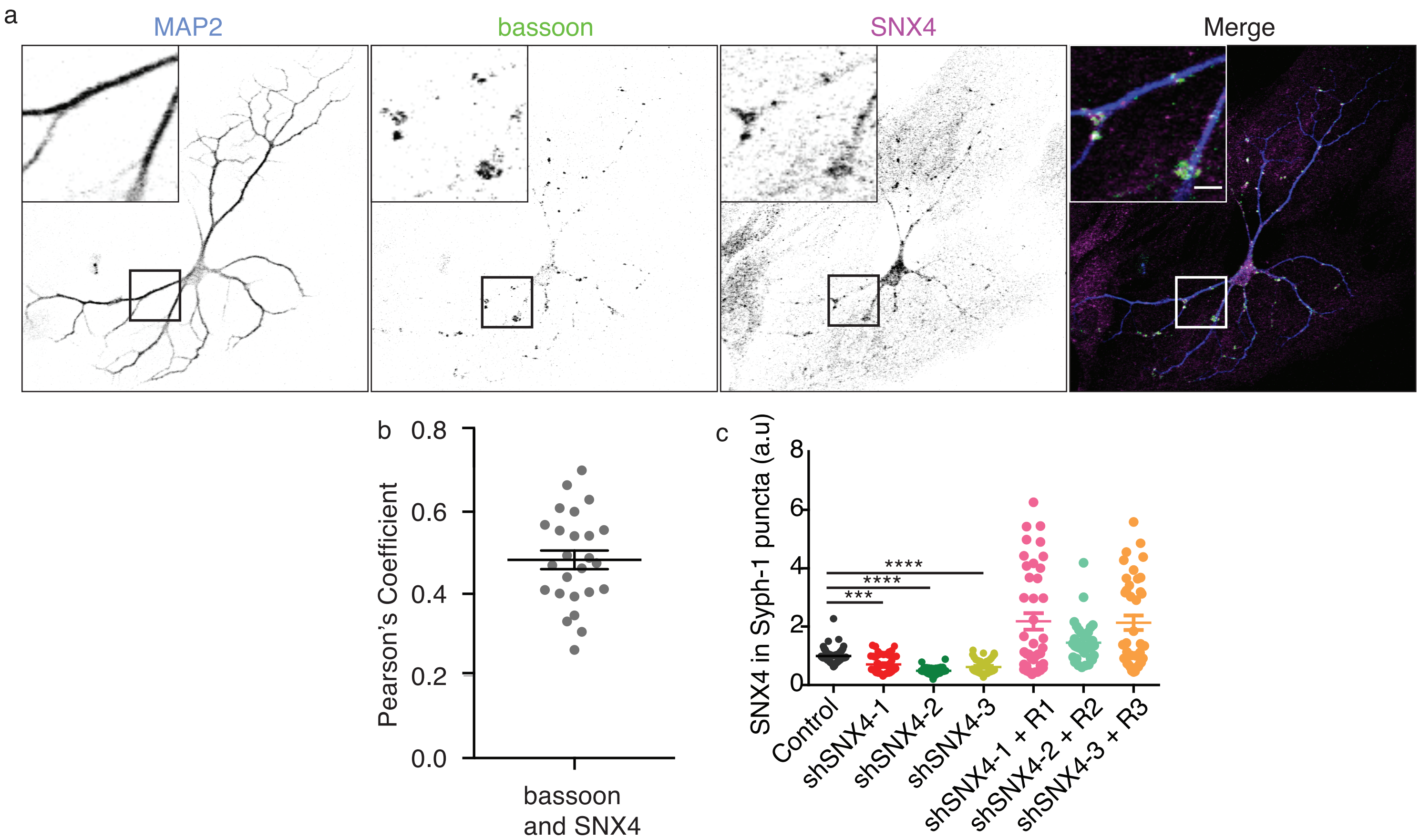

Supplementary Figure S3: SNX4 localizes at synapses. (a) Confocal microscopy of a hippocampal neuron on an astrocyte island immunolabelled with MAP2 (blue), bassoon (green) and SNX4 (magenta). Scale bar of the neuron image=20 µm, of the zoomed neurites=5 µm. (b) Pearson's coefficient for the co-localization between bassoon and SNX4 in neurites. (c) Quantification of Figure 1d data of SNX4 staining intensity in synaptophysin-1 puncta relative to control. Detailed information (average, SEM, n and statistics) is shown in Supplementary Table S1.

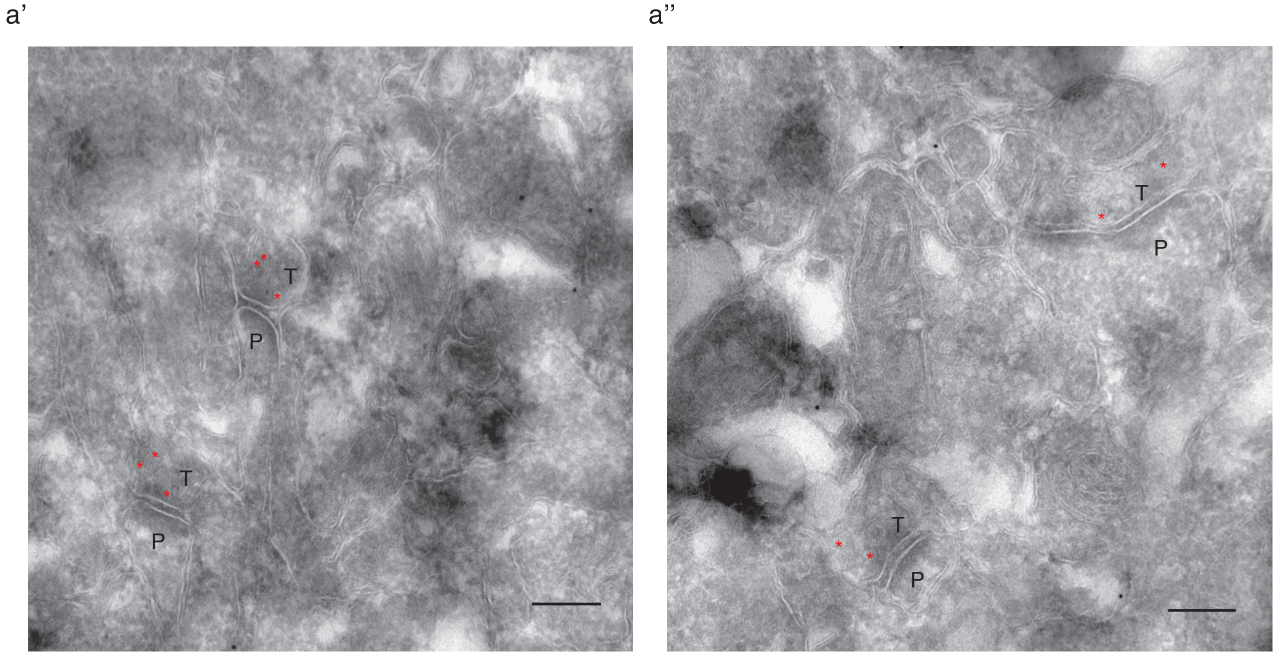

Supplementary Figure S4: Electron micrographs of the negative controls for immuno-gold labelling against SNX4 (a’, a’’). Negative control processed in parallel with the immunolabelling for SNX4 but preincubating the primary antibody with the blocking peptide (Synaptic Systems, Cat. No. 392-0P at a ratio of 1:10). ‘P’ indicates postsynaptic side, and ‘T’ the presynaptic terminal and ‘*’ is placed in the inside of some synaptic vesicles. Scale bar=200nm.

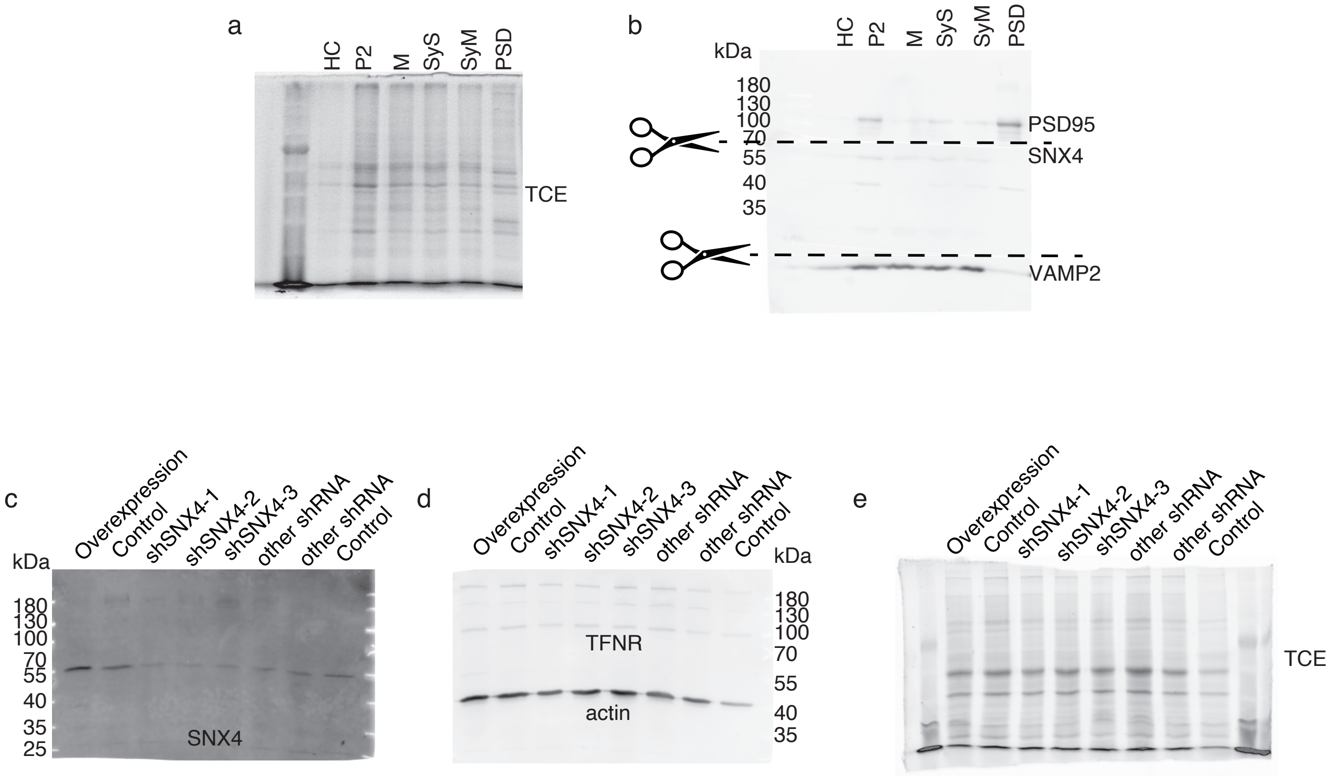

Supplementary Figure S5: Original uncropped blots. (a) Gel stained with TCE of the data shown in Figure 4 and (b) original uncropped blots for PSD95, SNX4, and VAMP2/Synaptobrevin-2. The first line is full hippocampal lysate from which the subcellular fractions were obtained. The membrane was cut as indicated by 'scissors and dash line' and each piece was incubated with the indicated primary antibody.Original uncropped western blot of the data shown in Figure 5a stained for SNX4 (c) and for (d) TFNR and actin and (e) gel stained with TCE.

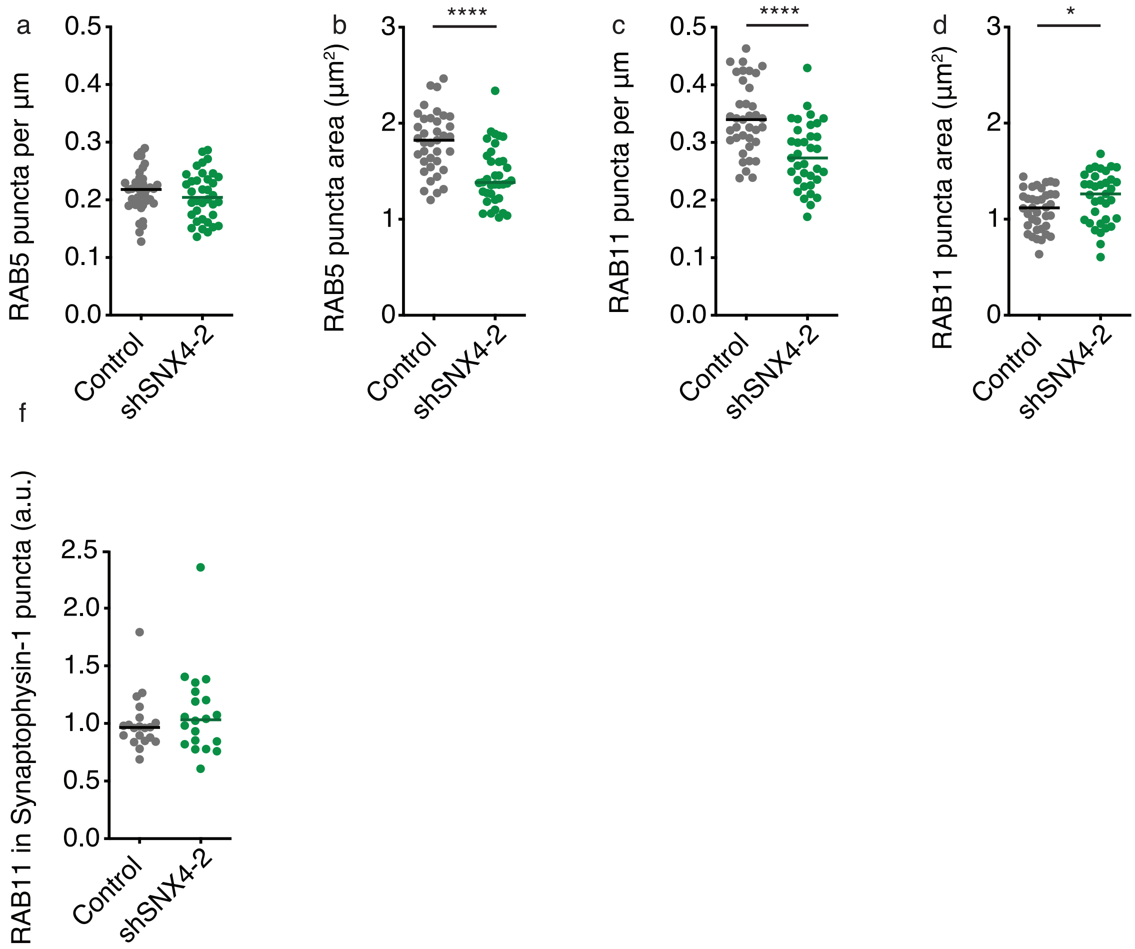

Supplementary Figure S6: RAB5 and RAB11 puncta density, area and intensity in synaptophysin-1 puncta upon SNX4 depletion. Quantification of Figure 2a data of RAB5 (a) number of puncta per µm (b) area of puncta. Quantification of Figure 2d data of RAB11 (c) number of puncta per µm (d) area of puncta. (f) Quantification of Figure 5d data of RAB11 staining intensity in synaptophysin-1 puncta normalized to the total RAB11 intensity in the neurites and relative to control. Detailed information (average, SEM, n and statistics) is shown in Supplementary Table S1.

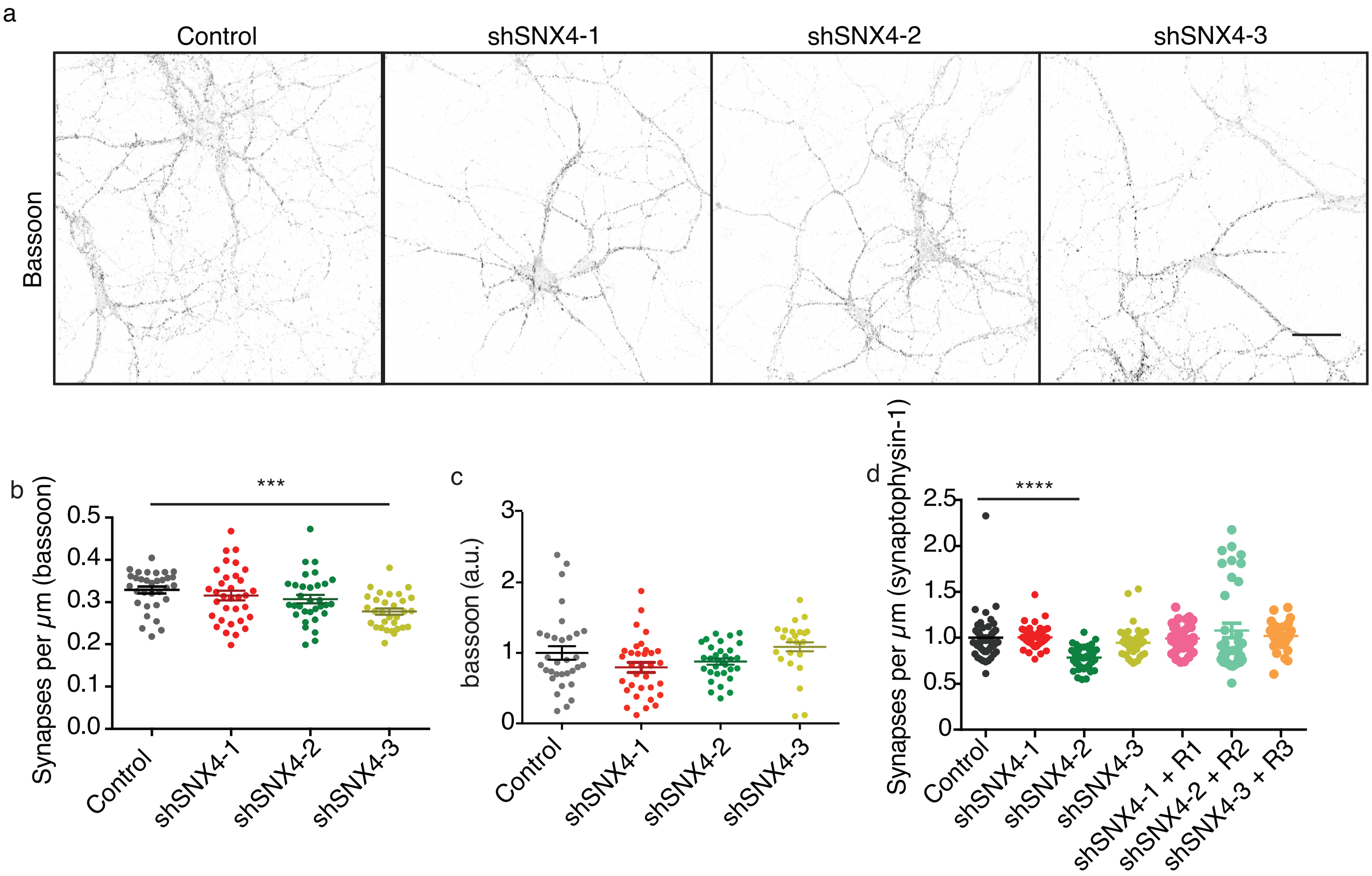

Supplementary Figure S7: SNX4 depletion does not affect synaptic density. (a) Confocal microscopy images of hippocampal neurons expressing control and SNX4 shRNAs immunolabelled with bassoon. Scale bar=40 μm. Quantification of (b) synaptic density using bassoon puncta and (c) total bassoon intensity relative to control (n=32±1 fields of view, N=3 cultures). (d) Quantification of synaptic density using synaptophysin-1 puncta relative to control from Figure 1d data. Detailed information (average, SEM, n and statistics) is shown in Supplementary Table S1.

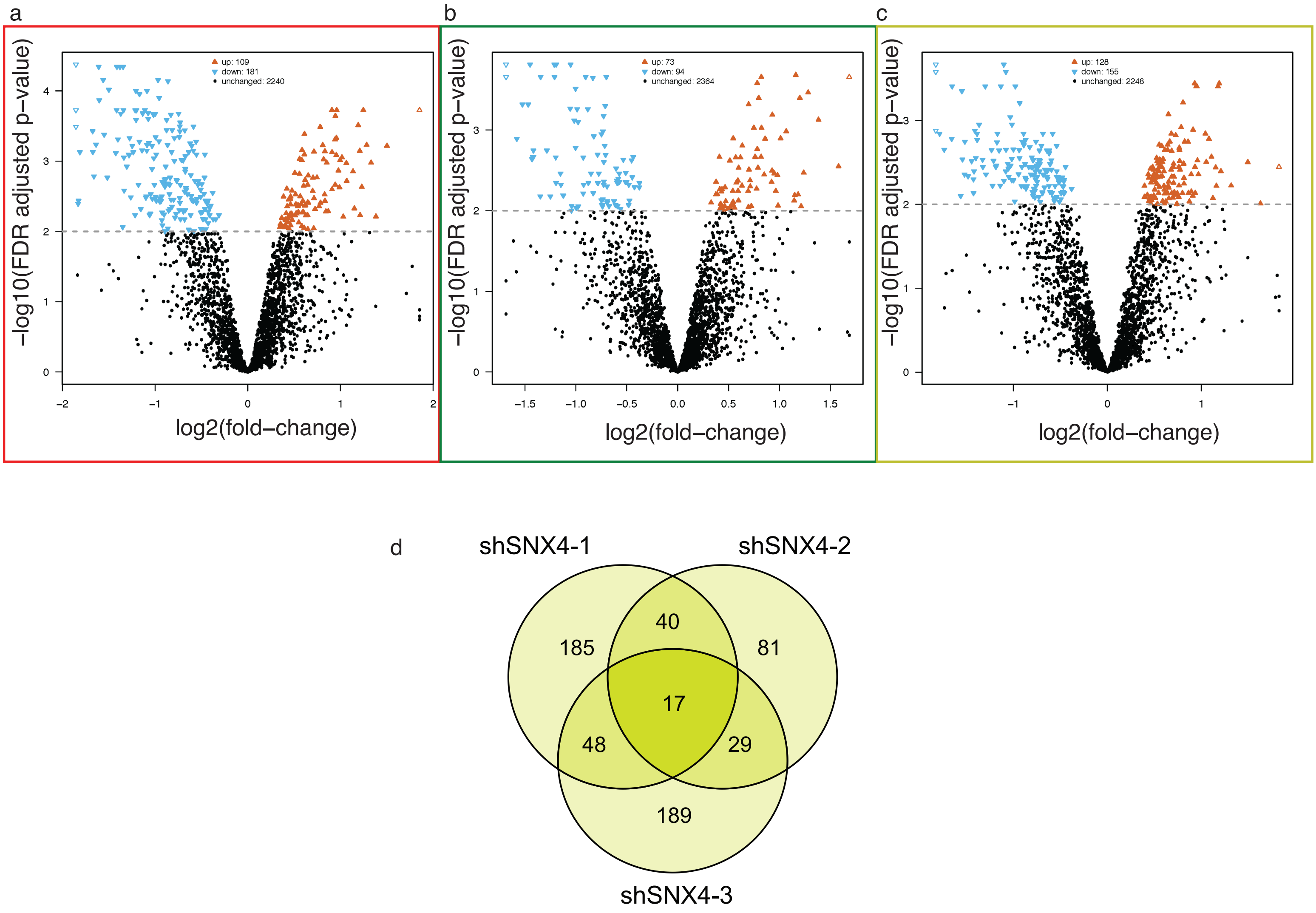

Supplementary Figure S8: Volcano plots showing the distribution of protein expression in cortical neurons expressing shRNA against SNX4 compared with control neurons. shSNX4-1 (a), shSNX4-2 (b), and shSNX4-3 (c). (d) Venn diagram showing the overlap among the dysregulated proteins in neurons containing shRNA against SNX4 compared with control.

Gene name	Description	foldchange(log2)		
		shSNX4-1	shSNX4-2	shSNX4-3
SNX4	Sorting nexin-4	-1.998****	-2.301****	-1.653****
SLC1A2	Excitatory amino acid transporter 2	-1.410****	-1.771****	-1.081****
GAD1	Glutamate decarboxylase 1	-1.414****	-1.526****	-1.153***
SCN2A1	Sodium channel protein	-1.898****	-0.997***	-1.445***
TTYH1	Protein tweety homolog 1	-0.893***	-0.726***	-1.400***
HSD17B7	3-keto-steroid reductase	-0.874****	-0.928***	-1.103****
COX6B1	Cytochrome c oxidase subunit 6B1	-0.864****	-0.596***	-1.329***
CALU	Calumenin	-0.827***	-0.446***	-1.102***
NSF	Vesicle-fusing ATPase	-0.463****	-0.583***	-0.446***
SARS	Serine--tRNA ligase, cytoplasmic	0.611*	0.431*	0.546*
GNS	N-acetylglucosamine-6-sulfatase	0.747***	0.850***	0.685***
CTSD	Cathepsin D	0.941****	0.723***	0.612***
WASL	Neural Wiskott-Aldrich syndrome protein	2.097****	2.559***	2.624***
GRIA1	Glutamate receptor 1	-0.994**	-0.315	-0.877**
SYT1	Synaptotagmin-1	-0.552**	-1.210***	-0.158
CADPS	Isoform 4 of Calcium-dependent secretion activator 1	-0.510*	-1.056****	-0.569**
GAD2	Glutamate decarboxylase 2	-0.996***	-0.656*	-0.708*
LAMP1	Lysosome-associated membrane glycoprotein 1	0.960***	0.438*	0.858**

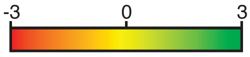

Supplementary Figure S9: Heatmap of the protein expression of dysregulated proteins in SNX4 knock down neurons. The log2 of fold change is color coded: Red indicates the the fold change of the maximum downregulation, green indicates the maximum upregulation and yellow no dysregulation. Among the 134 significantly regulated proteins in at least 2 shRNAs, the 9 down-regulated protein in all knockdown groups were SNX4, SLC1A2, GAD1, SCN2A1, TTYH1, HSD17B7, COX6B1, CALU, NSF and the up-regulated were SARS, GNS, CTSD and WASL. Upon SNX4-targetted shRNA expression GRIA1, SYT1 and CADPS were dysregulated synaptic proteins and CTSD and LAMP1 lysosomal proteins. When FDR corrected P-values were lower than 0.05, significance was noted in the figure as: *P<0.05, **P<0.01, ***P<0.001, ****P<0.0001.

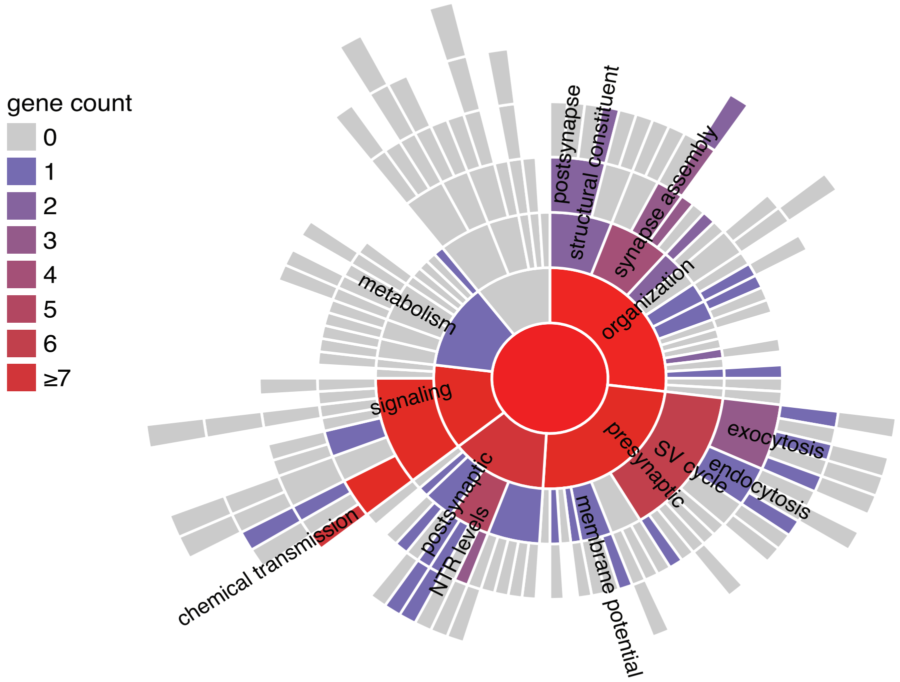

Supplementary Figure S10: Sunburst plot showing the annotation in synaptic functions of the altered proteins in the same direction in at least 2 shRNA against SNX4 expressing neurons (biological processes SynGO terms). Inner rings are parent terms of more specific child terms in the outer rings, colour coded according to gene count in each term. (Output plot of SynGO 1.0 dataset version/release: 20180731)**.**
